# Molecular Genetics of Glaucoma: Subtype and Ethnicity Considerations

**DOI:** 10.3390/genes12010055

**Published:** 2020-12-31

**Authors:** Ryan Zukerman, Alon Harris, Alice Verticchio Vercellin, Brent Siesky, Louis R. Pasquale, Thomas A. Ciulla

**Affiliations:** 1 Department of Ophthalmology, Icahn School of Medicine at Mt. Sinai, New York, NY 10029, USA; ryan.zukerman@gmail.com (R.Z.); alon.harris@mssm.edu (A.H.); alice.verticchio@mssm.edu (A.V.V.); brent.siesky@mssm.edu (B.S.); louis.pasquale@mssm.edu (L.R.P.); 2 Department of Ophthalmology, University of Miami Miller School of Medicine, Miami, FL 33136, USA; 3 Glaucoma Unit, Istituto di Ricovero e Cura a Carattere Scientifico (IRCCS) G. B. Bietti Foundation for Study and Research in Ophthalmology, 00198 Rome, Italy; 4 Department of Ophthalmology, University of Pavia, 27100 Pavia, Italy; 5 Vitreoretinal Medicine and Surgery, Midwest Eye Institute, Indianapolis, IN 46290, USA

**Keywords:** glaucoma, genetics, genome-wide association study, genetic/polygenic risk score, primary open-angle glaucoma, exfoliation syndrome, exfoliative glaucoma, primary angle-closure glaucoma

## Abstract

Glaucoma, the world’s leading cause of irreversible blindness, is a complex disease, with differential presentation as well as ethnic and geographic disparities. The multifactorial nature of glaucoma complicates the study of genetics and genetic involvement in the disease process. This review synthesizes the current literature on glaucoma and genetics, as stratified by glaucoma subtype and ethnicity. Primary open-angle glaucoma (POAG) is the most common cause of glaucoma worldwide, with the only treatable risk factor (RF) being the reduction of intraocular pressure (IOP). Genes associated with elevated IOP or POAG risk include: *ABCA1*, *AFAP1*, *ARHGEF12, ATXN2*, *CAV1*, *CDKN2B*-*AS1*, *FOXC1*, *GAS7*, *GMDS*, *SIX1*/*SIX6*, *TMCO1*, and *TXNRD2*. However, there are variations in RF and genetic factors based on ethnic and geographic differences; it is clear that unified molecular pathways accounting for POAG pathogenesis remain uncertain, although inflammation and senescence likely play an important role. There are similar ethnic and geographic complexities in primary angle closure glaucoma (PACG), but several genes have been associated with this disorder, including *MMP9*, *HGF*, *HSP70*, *MFRP*, and *eNOS*. In exfoliation glaucoma (XFG), genes implicated include *LOXL1*, *CACNA1A*, *POMP, TMEM136, AGPAT1, RBMS3,* and *SEMA6A*. Despite tremendous progress, major gaps remain in resolving the genetic architecture for the various glaucoma subtypes across ancestries. Large scale carefully designed studies are required to advance understanding of genetic loci as RF in glaucoma pathophysiology and to improve diagnosis and treatment options.

## 1. Introduction

Glaucoma is the world’s leading cause of irreversible blindness, implicated in approximately 12% of cases globally [1,2]. Glaucoma represents a degenerative optic neuropathy characterized by the progressive degeneration of retinal ganglion cells and the retinal nerve fiber layer (RNFL), which leads to corresponding visual field defects. While the major risk factor (RF), and only modifiable RF, for disease onset and progression is an elevated intraocular pressure (IOP) [3], the pathogenesis of the disease is both multifactorial and still poorly understood [4,5,6,7].

Importantly, glaucoma may be classified in different specific subtypes with different pathophysiologic mechanisms, including primary open-angle glaucoma (POAG), primary angle-closure glaucoma (PACG), and secondary exfoliation glaucoma (XFG), which have all been shown in the literature to be more prevalent in populations of different races [8,9,10,11]. Specifically, POAG, which is characterized by increased resistance to aqueous fluid outflow through the trabecular meshwork, has been shown to be more prevalent, to present earlier, and to be more severe in patients of African descent (AD) than patients of European descent (ED). By contrast, PACG, which is caused by blocked access to the outflow tracts, is well documented to be more prevalent in populations of Asian descent [2,12,13,14,15,16,17,18,19,20,21,22,23,24,25,26]. Meanwhile, XFG, a common form of secondary glaucoma which is a sequela of exfoliation syndrome (XFS), has an interesting ethnic and geographic distribution [27].

In recent years, genetic and genomic studies have shown encouraging evidence for a possible genetic contribution to the pathogenesis of glaucoma. Starting with studies of single-gene variants, genetic analysis has expanded to whole exome sequencing (WES), genome-wide association studies (GWAS), and genetic/polygenic risk scoring (G/PRS) as methods to illuminate the possible genetic RFs underlying glaucoma [28,29]. These studies have largely painted a picture of glaucoma as a disease that follows a complex inheritance pattern, indicating the importance of underlying genetic variants as a key to disease pathogenesis [28].

In summary, glaucoma is a complex disease, with different types and presentations, and ethnic and geographic disparities among different populations. These features complicate the genetic study of glaucoma. It is crucial to understand the natural history among different ethnic groups, in order to identify genetic RFs that have the potential to transform the way that glaucoma is screened, diagnosed, and treated worldwide. Therefore, this review aims to synthesize the current literature on glaucoma and genetics, as stratified by glaucoma subtype and ethnicity.

## 2. Materials and Methods

PubMed, Embase, Ovid, Scopus, and Trip searches were conducted through 1 December 2020 to evaluate all pertinent articles, abstracts, and ongoing research projects. Searched key words include: glaucoma, genetics, genome-wide association study, genetic/polygenic risk score, primary open-angle glaucoma, exfoliation syndrome, exfoliative glaucoma, primary angle-closure glaucoma, African descent, European, Asian, Middle Eastern, Latin American, Hispanic, and genes. The same key words were used in all search software. Articles were screened for relevance and analyzed based on inclusion criteria, population, and specific genes studied. Data was collected and organized using Microsoft Word (version 16.30), Microsoft Excel (version 16.30), and EndNote (X8.2).

References from all relevant articles found were reviewed to ensure inclusion of all relevant articles. 

## 3. Discussion

### 3.1. Primary Open-Angle Glaucoma

Primary open-angle glaucoma (POAG) is the most common cause of glaucoma worldwide, accounting for approximately 74% of all glaucoma cases [1]. As the name suggests, POAG is a form of glaucoma characterized anatomically by an open angle. The angle of the eye is the junction of the cornea and the iris and is physiologically significant as the site where the aqueous humor of the eye drains out of the anterior chamber through the trabecular meshwork (responsible for approximately 90% of aqueous outflow) and into Schlemm’s canal, before draining into the venous system. Therefore, POAG is, by definition, a form of glaucoma where drainage of aqueous humor is obstructed despite the anatomy of the angle allowing for aqueous drainage [30].

The major RF, and only treatable RF, for POAG is an elevated IOP while, other RFs including older age, family history (specifically siblings and parents), ethnicity (AD), thin central corneal thickness (CCT), and impaired ocular blood flow all may also play a role [30]. Interestingly, although IOP appears to play a role in the pathogenesis of POAG via-mechanical strain on the lamina cribrosa, not all forms of POAG present with elevated IOP [30]. Importantly, normal tension glaucoma (NTG) represents a subtype of POAG characterized by glaucomatous optic neuropathy in the setting of normal IOP. It has been suggested that NTG may be the result of low cerebrospinal fluid pressure that creates a similar mechanical stress effect on the optic nerve as opposed to the elevated IOP that presents in classic POAG [31,32].

Previous studies have demonstrated that POAG disproportionately impacts patients of AD when compared to patients of ED [12,13,14], and research into these disparities has shown that AD patients have a significantly stronger vascular component to the pathophysiology of their glaucoma than ED patients, as well as a statistically significant decrease in retrobulbar blood flow [6,7]. Underlying these differences, however, are complex genetic RFs that contribute to disease pathogenesis, progression, and severity.

In order to identify the genetic RFs for POAG, researchers utilize genome-wide association studies (GWAS) to analyze groups of patients with POAG for common genetic variants. After these variants—single nucleotide polymorphisms (SNPs)—are identified, further analysis associates them with disease. GWAS are challenged by the fact that associated variants cannot be proven to be causative in the disease process [33]. Additionally, in the case of a disease with a complex inheritance pattern like POAG, single genetic variants may have limited value in assessing risk.

Given these limitations, recent research has focused on reports of polygenic risk scores (PRS) [34]. Although PRS similarly cannot point to mechanistic causes of disease, these studies are better suited to quantifying risk of disease and disease progression by analyzing multiple genetic variants at once [35].

In the case of POAG, most GWAS have been performed in populations of ED and Asian descent, leaving a gap in knowledge surrounding AD populations, which are disproportionately impacted by disease [8]. This knowledge gap is exacerbated by the fact that European-derived PRS generally do not replicate in non-European studies [34,36]. Therefore, extensive research is warranted to assess the genetic basis of POAG in different ethnicities.

#### 3.1.1. European Descent

In the past decade, several genes have been identified through single-gene analysis and GWAS as associated with POAG and a variety of endophenotypes for disease. For example, genes associated with IOP include: *ABCA1*, *AFAP1*, *ARHGEF12*, *ATXN2*, *CAV1*, *CDKN2B*-*AS1*, *FOXC1*, *GAS7*, *GMDS*, *SIX1*/*SIX6*, *TMCO1*, and *TXNRD2* [8,37,38,39,40,41,42] (Table 1).

In 2018, MacGregor, et al. reported a combined analysis of 103,914 participants from the UK Biobank with 101 SNPs for IOP identified by the International Glaucoma Genetic Consortium (*n* = 29,578). They found 53 SNPs with evidence of association among 11,018 glaucoma cases and 126,069 controls, as well as an additional 22 independent genes associated with IOP [68]. Using this data, MacGregor, et al. created an allele score (PRS) for a cohort of 1734 patients with advanced glaucoma and 2938 controls. They demonstrated an increased risk (odds ratio (OR): 5.6; 95% confidence interval (CI): 4.1–7.6) of glaucoma for participants with higher allele scores.

Given the high number of risk alleles for POAG, studies similar to this study by MacGregor, et al. have become the gold standard for quantifying genetic data. In fact, a 2018 meta-analysis from Khawaja, et al. identified 112 loci, including 68 novel loci, associated with IOP and the development of POAG, and concluded that genetic prediction models likely play a role in the future of POAG screening and treatment [69]. This meta-analysis used data from the Glaucoma Genes and Environment (GLAUGEN) study (a part of the Gene Environmental Association Studies consortium) [70], and the National Eye Institute Glaucoma Human Genetics Collaboration (NEIGHBOR) study.

More recently, in 2019, Fan, et al. created a cross-sectional study also including European white individuals with POAG and controls from the GLAUGEN study and the NEIGHBOR study [51]. They created a PRS that included risk variants from the 12 previously identified genes associated with POAG: *ABCA1*, *AFAP1*, *ATXN2*, *CAV1*, *CDKN2B-AS1*, *FNDC3B, FOXC1*, *GAS7*, *GMDS*, *SIX1/SIX6*, *TMCO1*, and *TXNRD2*. They discovered that the PRS was significantly associated with POAG (OR per 1-point increase in score = 1.24; 95% CI: 1.21–1.27; *p* = 3.4 × 10^−66^), as well as an earlier age at diagnosis (β = −0.36; 95% CI: −0.56 to −0.16; *p* = 4.0 × 10^−4^) [71]. 

Similarly, in 2019, Gao, et al. constructed several PRSs for IOP to assess the association between IOP and POAG, using data from 110,964 European UK Biobank participants and >1200 SNPs [72]. This study used a considerably larger number of SNPs than other studies and demonstrated that the PRS was significantly associated with both IOP (*p* ~ 10^−200^) and POAG (*p* = 1.8 × 10^−77^). Additionally, they noted that patients with higher IOP PRS had a 6.34 (95%CI: 4.82-8.33; *p* = 2.1 × 10^−57^) times higher risk of having POAG [72]. Meanwhile, in 2020, Qassim, et al. created a similar PRS of IOP-associated SNPs and evaluated the PRS among patients diagnosed with POAG. They found that the IOP PRS was significantly associated with a higher maximum IOP (1.7 mmHg; standard deviation (SD) 0.62 mmHg; *p* = 0.006), earlier age at diagnosis (3.7 years, SD 1.0 years; *p* < 0.001), more family members affected (0.46 members, SD 0.11 members; *p* < 0.001), and higher rates of incisional surgery (OR 1.5; 95%CI: 1.1–2.0; *p* = 0.007) [73]. 

GWAS and PRS have been used in other types of studies about POAG. In 2018, Iglesias, et al. tested myopia PRSs with POAG and a variety of POAG endophenotypes—specifically vertical cup-disc ratio (VCDR), cup area, IOP, and RNFL thickness. They found no association between PRS of myopia and POAG (*p* = 0.81), VCDR (*p* = 0.42), cup area (*p* = 0.25), IOP (*p* = 0.07), or RNFL thickness (*p* = 7.7 × 10^−3^), demonstrating that there is no evidence for overlap of genetic RFs between POAG and myopia [46]. Additionally, a 2017 PRS from a southern European Mediterranean population demonstrated a strong association between a PRS of 4 SNPs and POAG (OR: 2.92; 95%CI: 1.79–4.77, *p* < 0.001), while also showing an inverse correlation between the PRS and low levels of vitamin C (*p* = 0.002) and vitamin E (*p* = 0.001), suggesting a possible role for oxidative stress mechanisms in the pathogenesis of POAG [74].

Alternatively, it is important to note that several genes (*MYOC*, *OPTN*, WD repeat domain 36) have been previously associated with an autosomal dominant form of POAG, although less than 10% of cases are associated with these genes [30]. *MYOC*, specifically, is implicated in approximately 4% of POAG cases, but it is also closely associated with juvenile open-angle glaucoma [30]. Researchers have found close to 100 *MYOC* SNPs associated with disease, and it is suggested that mutations lead to accumulation of misfolded myocilin, leading to elevated IOP [30,75]. Alternatively, *OPTN* and *TBK1* autosomal dominant mutations are associated with NTG, accounting for between 2–3% of all NTG cases [76,77]. 

Still, familial modes of inheritance allow for other forms of genetic study. For example, in 2018, WES was used to study a large POAG family in the Netherlands. Researchers identified a variant in *TP53BP2* that was associated with POAG in this family when compared to population-matched controls but were unable to demonstrate a dominant or recessive inheritance pattern. They noted, however, that previously *TP53BP2* had been associated with apoptosis regulation in retinal ganglion cells, suggesting a possible mechanism for POAG in this family [78].

Finally, it is important to note that researchers have found genetic loci specifically associated with NTG in ED populations. In 2009, Wolf et al. conducted a case-control study in the German population to find genetic risk factors for NTG. They found five candidate genes to be unlikely to confer risk of NTG (*RDX*, *SNX16*, *OPA1*, *SOD2*, and *CYP1B1*) while four others showed a trend toward association (*OPTN*, *MFN1*, *MFN2*, and *PARL*), ultimately concluding that these four genes warranted further study [79].

In 2012 Wiggs et al. conducted a GWAS that found two genetic loci were specifically associated with NTG in a meta-analysis of GLAUGEN and NEIGHBOR data: 9p21 containing *CDKN2B-AS1* (OR 0.58, 95%CI: 0.50–0.67, *p* = 1.17 × 10^−12^) and a region on 8q22 (OR 0.62, 95%CI: 0.53–0.72, 8.88 × 10^−10^) [41]. According to the authors, these loci are associated with transforming growth factor beta (TGF-β) signaling, so they performed a genomic pathway analysis showing NTG was associated with the TGF-β pathway, suggesting that TGF-β may generally contribute to glaucomatous optic neuropathy independent of IOP [41]. These associations were later confirmed by Bailey et al., who also suggested that the association between *CDKN2B-AS1* and NTG may in fact be stronger than the association with POAG overall (OR 1.6 vs. OR 1.4) [38]. Additionally, their meta-analysis of NTG cases found a novel locus on chromosome 12q associated with NTG (OR 1.48, *p* = 2.41 × 10^−8^), but was not significant on the genome-wide level when analyzed datasets were combined [38].

More recently, in 2017 Burdon, et al. conducted a retrospective case-control study which confirmed the association between NTG and the *CDK2NB* promoter in females in an Australian cohort (*p* = 0.001), confirming earlier studies that suggested female sex as a strong RF for NTG in ED populations [52,80]. Meanwhile, a 2015 analysis of the *ARHGEF12* gene in the Rotterdam Study population showed that *ARHGEF12* was associated with NTG (OR 1.29, *p* = 4.23 × 10^−2^) though to a lesser degree than high-tension glaucoma (OR 1.66, *p* = 2.81 × 10^−9^) [42]. Additionally, studies in ED populations between NTG and endothelin-1 gene polymorphisms have shown no association with disease nor with specific RF [81,82,83,84].

#### 3.1.2. African Descent

Populations of AD are particularly burdened by POAG. In fact, the prevalence of POAG in AD is almost double that of ED populations, and is particularly high in West Africans. Additionally, POAG risk is up to 5 times higher for AD individuals, who are also more likely to have severe cases of POAG that may result in total blindness [8]. Despite this high disease burden, specific genetic markers and their influence of POAG pathology are not well-defined by current genetic studies within AD populations. This is complicated by differing effect size for loci in AD, and the fact that POAG genes identified in Caucasians are often unshared with persons of AD. Indeed, recent studies haven’t been able to replicate most of the genetic findings from ED and Asian GWAS suggesting private genetic variants in AD populations have yet to be identified [53,85,86,87]. In one example, Hauser, et al. conducted a GWAS among patients of African ancestry, but included individuals from Saudi Arabia in the analysis as individuals with African ancestry admixture. In this study they demonstrated that *APBB2* rs59892895T > C was associated with POAG (OR 1.32, 95%CI: 1.20–1.46, *p* = 2 × 10^−8^). Additionally, they noted that this SNP was only present in AD populations, and had a frequency <0.1% in populations of ED or Asian ancestry [48].

During the last decade, the African Descent and Glaucoma Evaluation Study (ADAGES) has tried to address the underlying causes and heightened risk and severity of POAG in the AD population. In 2009, the baseline data from ADAGES outlined many of the RFs that could help explain the higher prevalence of POAG in this population, including CCTs (*p* < 0.001), higher rates of diabetes mellitus (*p* < 0.001) and hypertension (*p* < 0.001), lower rates of heart disease (*p* = 0.001). Interestingly, ADAGES baseline data demonstrated no differences in mean IOP between populations (*p* = 0.79) [88].

In 2019, ADAGES III aimed to specifically describe the genetics of POAG in a comparative analysis between AD POAG populations and ED POAG populations and, more specifically, to find a genetic explanation for the phenotypic differences between the populations that researchers had noted in ADAGES I and II [89]. ADAGES III identified a novel genetic locus, *EN04*, which was associated with POAG in the AD population. Researchers also found a novel SNP associated with CDKN2B which suggested that there were different SNPs associated with POAG in ED (rs2393204) and AD (rs79721419) populations. Meanwhile, SNPs for *FNDC3B* and *GAS7* were identified in AD populations, but were also suggested to contribute to POAG in ED populations in addition to SNPs previously identified [29].

Importantly, ADAGES III also included 3 PRSs: one with 11 previously well-identified SNPs for POAG in ED and AD, and two variations with almost 13,000 SNPs that had shown an association with POAG in AD. PRS #2 (Area under the curve (AUC) = 0.74) and PRS #3 (AUC = 0.94) demonstrated a greater AUC value than PRS #1 (AUC = 0.62), suggesting that there are more SNPs that enhance POAG risk in AD than just the 11 previously well-identified SNPs. However, researchers were quick to note that these PRS require confirmation studies and clinical trials, so these PRS should not be used clinically [29]. 

In 2018, Bonnemaijer, et al. conducted the first POAG GWAS of continental Africans, using data from the Genetics in Glaucoma patients from African descent study. They confirmed three POAG loci that were previously demonstrated in ED: *CDKN2B-AS1*, *TMCO1*, and *TXNRD2*. Additionally, they found an SNP (rs141186647) at a novel locus as well, *EXOC4* (OR 0.48; *p* = 3.75 × 10^−8^) [53]. Interestingly, a previously identified SNP (rs1063192) near *CKDN2B-AS1* that had been reported in Afro-Caribbean populations in Barbados was unable to be replicated [53,85]. It is important to consider the admixture of Caucasians in Barbados that may be responsible for this differential finding, as SNP (rs1063192) is monomorphic within the AD population. Finally, a PRS was calculated based on 15 known ED/AD SNPs and showed a significant association with POAG (*p* = 2.81 × 10^−5^) [53].

A separate 2018 GWAS of a multiethnic cohort from the Genetic Epidemiology Research in Adult Health and Aging (GERA) cohort identified five novel POAG loci which were replicated using a UK Biobank cohort. Importantly, when stratified by ethnicity, researchers found a significant association between African ancestry and increased POAG risk (*p* = 0.01), and approximately 3.1% of the variance in POAG risk in African-Americans could be explained by newly identified SNPs compared to 0.5% of the variance attributed to previously discovered genetic variants [45]. 

As for NTG specifically, in 2011 Fingert, et al. reported a novel locus for NTG on chromosome 12q14 designated GLC1P that contained the gene *TBK1* and was associated with African American pedigrees of NTG. Further analysis demonstrated that a 780 kbp duplication in the locus was co-inherited with NTG throughout the pedigree (maximum non-parametric linkage score = 19.7, max LOD score = 2.7) [64]. Further analysis of this duplication using real-time PCR demonstrated that the genes within the duplication (*TBK1, XPOT*, *RASSF3* and *GNS*) were all expressed within the retina, suggesting a possible association with NTG and warranting further study to confirm association with either POAG or NTG specifically [64]. Other data specifically associating genetics with NTG in AD populations is lacking.

#### 3.1.3. Asian Descent

In Asian populations the disease burden of PACG is considerably higher than POAG, though POAG is still prevalent. In 2019 the Chinese Glaucoma Study Consortium (CGSC), the first national glaucoma database in China, published data of 10,892 patients (complete data for 5762 patients). While PACG was most prevalent (4588, 79.63%), POAG still made up a considerable portion of the glaucoma population in China (1116, 19.37%) [90].

In previous studies of the POAG-susceptibility loci previously associated with ED populations, several have also been associated with Asian populations including *ABCA1*, *CAV1, CDKN2A-CDKN2B*, *GAS7, PMM2*, *TMCO1,* and *SIX6*, while *TGFβR3* has repeatedly been associated with POAG in Asian populations [43,44,50,65]. In 2018, Shiga, et al. conducted a GWAS and replication study of 7378 Japanese POAG cases and 36.385 controls. They identified 11 POAG-associated loci: 4 known (*ABCA1, AFAP1, CDKN2B-AS1,* and *SIX6*) and 7 novel (*ANKRD55-MAP3K1, FNDC3B, HMGA2, LHPP, LMX1B, LOXL1,* and *MEIS2*) (*p* < 5.0 × 10^−8^) [47]. Of these seven, three single gene variants were also associated with POAG when analyzed in a Chinese population, and four in an ED population [8,47]. A 2009 GWAS from Nakano, et al. identified three POAG-associated susceptibility loci, but replication in other populations, specifically Middle Eastern populations, was unsuccessful [62]. 

Interestingly, several genetic loci have also specifically been associated with NTG in Asian populations. In 2010, the Writing Committee for the NTG Genetic Study Group of the Japan Glaucoma Society identified SNPs of *SRBD1* (OR 2.80, *p* = 2.5 × 10^−9^) and *ELOVL5* (OR 1.69, *p* = 4.1 × 10^−6^) as being associated with NTG in a cohort of 305 Japanese NTG patients and 355 controls. They suggested that *SRBD1* and *ELOV5* are involved in apoptotic mechanisms, so alterations to the regulation of these genetic pathways may be important in understanding the mechanism of NTG pathogenesis [56]. Interestingly, the role of the *SRBD1* gene appears to be unclear as a 2020 study from Jung, et al. showed no association between NTG and several *SRBD1* SNPs in a Korean cohort [91].

Meanwhile, in 2012 Takamoto, et al. confirmed the association between NTG and SNPs of the *CKDN2B* gene on chromosome 9p21 in a Japanese population (OR 2.00, 95%CI: 1.55–2.58, *p* = 7.40 × 10^−8^) [54]. More recently, in 2020, Lu, et al. conducted an analysis to clarify the relationship between the *CAV1-CAV2* locus and specifically NTG. They determined that rs4236601, which was previously identified in GWAS of Chinese POAG patients, was significantly associated with NTG in two different Chinese cohorts (OR 4.55, p_meta_ = 0.0019, I^2^ = 64%) [49]. Additionally, a specific SNP of *MMP*-9, a gene more commonly associated with PACG, was shown to be significantly associated with NTG (*p* = 0.021), though of the five SNPs studied none of them were significantly associated with POAG (*p* > 0.05) [60]. 

In both Japanese and Korean populations, studies have shown association between *HK2* and *NCK2* genes with NTG. First, in Japan, a two-stage case-control study showed significant allelic association of rs678350 in the *HK2* gene (*p* = 4.7 × 10^−4^) and rs2033008 in the *NCK2* gene with NTG [92]. In the Korean population, however, study of the same SNPs showed only significance for the *HK2* SNP with a significantly higher minor allele frequency (MAF) in NTG patients (MAF = 0.32) than controls (MAF = 0.23) (OR 1.586, 95%CI: 1.144–3.180, *p* = 0.015) [93]. Both of these genes are expressed in the retinal ganglion cell layer, suggesting a possible mechanism for the associated glaucomatous degeneration [92]. On the other hand, several other genes have shown no association with NTG in Asian populations including *POU4F1*, *POU4F2*, and *ISL1*—all of which are associated with retinal ganglion cell development—as well as SNPs of the *ASB10* gene, *TLR4* gene, *TLR2* gene, and *SLC1A3* gene [61,94,95,96,97].

Asian studies have also examined genetic RFs for POAG endophenotypes. For example, in 2015 Tham, et al. calculated PRSs for a multiethnic Asian population in the Singapore Epidemiology of Eye Diseases Study. The PRSs contained risk alleles for IOP and VCDR, and found a higher PRS for each endophenotype to be significantly associated with a higher risk of POAG [IOP: OR 2.5, 95%CI: 1.54–4.012, *p* = 2.0 × 10^−4^; VCDR: OR 2.31, 95%CI: 1.50–3.55, *p* = 1.4 × 10^−4^)]. In fact, when patients were in the top tertile for both IOP and VCDR, they were 7.77 times more likely to have POAG, 95%CI: 3.03–19.93, *p* = 2.0 × 10^−5^) [98]. Meanwhile, in 2017, a PRS of Japanese patients evaluated IOP, VCDR, in high tension glaucoma (HTG) and NTG. Using nine IOP-related SNPs, the study demonstrated that an increased PRS led to significant increases in maximum IOP (*p* = 0.012) and VCDR (*p* = 0.010) and a 2.54 times higher risk of HTG (*p* = 0.0085, Chi-square test). Researchers concluded that IOP-related single-gene variants may have an additive effect on IOP and VCDR, and that there may be genetic differences between HTG and NTG, suggesting that POAG phenotype (HTG/NTG) may be dependent on IOP-related genetic variants [99]. 

Lower CCT has also been associated with POAG in Japanese populations, and consequently researchers have looked for single gene variants associated with corneal structural development [8,46]. In 2018, Iglesias, et al. conducted a cross-ancestry GWAS of more than 25,000 participants both ED and Asian descent. They identified 19 loci associated with CCT, but found little data linking CCT SNPs and POAG risk, and only one variant, near *ADAMTS8*, associated with POAG when analyzing the Asian cohort alone. Ultimately, however, none of the single gene variants for CCT ultimately met a genome-wide significance threshold for POAG [8,46].

Interestingly, while research into CCT SNPs has found significant genomic association in Asian populations, association between CCT and POAG has not been as strong. A 2013 meta-analysis noted a significant association between *FNDC3B* and POAG (*p* = 5.6 × 10^−4^), while also noting that an allele near *FNDC3B* (rs4894535) associated with lower CCT actually led to a decreased POAG risk (OR 0.83, 95%CI: 0.74–0.92) [100]. Ultimately, they demonstrated that alleles associated with lower CCT showed an association with keratoconus and not with POAG. 

Finally, research has suggested that glutathione S-transferase (GST) polymorphisms may be associated with an increased risk of POAG. In 2013, a meta-analysis demonstrated that the GSTM1 null genotype was associated with an increased risk of POAG in Asian populations but not ED and mixed populations [101]. A separate 2013 met-analysis confirmed these findings, showing that the association between the GSTM1 null genotype and risk of POAG was not statistically significant in ED populations (OR 1.13, 95%CI: 0.69–1.84, *p* = 0.64) or Latin American populations (OR 1.09, 95%CI: 0.62–1.92, *p* = 0.77), but was statistically significant in an East Asian population (OR 1.41, 95%CI: 1.04–1.90, *p* = 0.026) [102].

#### 3.1.4. Middle Eastern Descent

There is very little information specifically focusing on POAG in Middle Eastern (ME) populations. In fact, there are only two published studies that specifically address POAG prevalence in the ME—and both use European models to estimate prevalence [1,103]. The Middle East, however, is a challenging region to study given the relative ambiguity of the region’s borders—generally accepted as West Asia and North Africa, but occasionally including Turkey, the South Caucuses, Afghanistan and Pakistan. 

As for genetics, the Middle East has a relatively high rate of consanguinity/endogamy, suggesting that genetics may play are role in specific subsets of this population [104]. Generally, however, follow-up of genetic loci that have been identified in other populations—specifically loci identified in a Japanese population—have led to largely negative associations in ME populations [62,105,106,107,108,109,110,111]. Individual SNPs for other genes, including the *SIX1/SIX6* locus and the endothelial nitric oxide synthase gene (*NOS3*), have been identified as associated with POAG in a Saudi Arabian population [63,112]. Meanwhile, in Iran, an association between POAG and the p53 pro72 allele (*p* < 0.05), as well as polymorphisms of the IL-10 gene promoter, have been noted and reflect findings in a Chinese population [113,114].

As mentioned previously in the AD population section, the GWAS conducted by Hauser, et al. included patients of AD mixed with individuals from Saudi Arabia identifying *APBB2* rs59892895T > C as associated with POAG in the AD/Saudi Arabian populations [48]. Currently there is a lack of large uniform genetic data on ME populations and POAG, with available data often segregated to specific countries within the ME or with admixture of other population genetics.

#### 3.1.5. Latin American Descent

Similar to the ME, Latin America is a complex region including people from North, South, and Central America. The genetics of people of Latin American descent (LAD) are similarly complex, as LAD ancestry includes European immigrants, African ancestry, and local indigenous populations [115].

POAG prevalence studies in LAD show a slightly higher prevalence rate than ED [8]. The Los Angeles Latino Eye Study evaluated a population of 6357 self-identified Latinos, mostly of Mexican ancestry, in California and found a prevalence of POAG to be 4.74% (95%CI: 4.22–5.30) and a prevalence of elevated IOP to be 3.56% (95%CI: 3.12–4.06%) [116]. Meanwhile, Proyecto VER assessed 4774 Hispanic adults in Arizona and found an OAG prevalence of 1.97% (95%CI: 1.58–2.36%), and noted this prevalence value was between reported prevalence values for ED and AD populations [117]. Interestingly, POAG prevalence per decade of age is increasing most in Hispanics (2.31, 95%CI 2.12–2.52) [118].

In 2013, Buentello-Volante., et al. conducted the first case-control study for POAG risk alleles in a Mexican population. Although the analysis showed the 26 risk variants were not associated with an elevated risk for POAG, SNP analysis of the CG genotype of rs5335 in *EDNRA* showed a protective effect (OR 0.5, 95%CI: 0.3–0.9, *p* = 0.03), as did a haplotype analysis of *CYP1B1* [119]. 

Prior to this study, POAG in LAD populations had generally only been studied in the context of single-gene variants, and this trend largely continues. In Brazil, a variety of studies have suggested the involvement of SNPs of *CDKN2B-AS1* [55,85], *eNOS* [57], *IL1A* and *IL1B* [59], and *TLR4* [66,67]. In fact, associations between the GSTM1 null polymorphism has also been associated with POAG in Brazilian populations [101,120,121]. Meanwhile, *MYOC* and *OPTN* polymorphisms have shown no association [122], although *MYOC* variants studied in Peruvian populations have shown both novel and previously reported genetic variants from other populations [123,124].

In 2018, the previously mentioned multi-ethnic GWAS from Choquet, et al. suggested association between rs9494457 of *PDE7B* and POAG in a LAD population (*p* = 0.005). They reported that analyzed SNPs in the GERA database could account for approximately 2.0% of variation in POAG risk in populations of LAD, while newly discovered SNPs increased this proportion to approximately 3.3% [45]. Ultimately, they concluded that the identified genetic loci were unable to account for ancestry effects in LAD populations, suggesting that more population-specific loci await discovery [45].

Finally, in 2018 Nannini, et al. conducted the first association study between PRS and VCDR in a LAD population. Using a combination of 68 VCDR SNPs, they analyzed two PRSs—one weighted toward ethnic-specific genetic variants, and one unweighted—they determined that both PRSs were significantly associated with VCDR (*p* < 0.0001), and accounted for approximately 2.7% of variation in VCDR. Additionally, when weighted (OR 1.75, 95%CI: 1.09–2.81, *p* = 0.0015) and unweighted (OR 2.00, 95%CI: 1.24–3.22, *p* = 0.0042), both PRSs were associated with significantly higher odds of POAG. Since using ethnic-specific genetic variants in the form of the weighted PRS improved the ability of the PRS to identify POAG (*p* < 0.0001), the authors suggested additional genetic variants that haven’t been reported may help improve the discriminatory ability of this PRS for POAG even more [125].

### 3.2. Primary Angle-Closure Glaucoma

Similar to POAG, PACG is associated with elevated IOP, however the key factor distinguishing PACG involves blockage of aqueous outflow through the trabecular meshwork in the angle of the eye [30]. There are several different mechanisms that may lead to angle closure, most commonly anatomic abnormalities such as pupillary block, plateau iris, choroidal thickness, and uveal expansion, but physiologic changes such as pupillary dilation from medication or low light may also cause an acute angle-closure glaucoma [126,127]. Importantly, though, angle closure generally leads to accumulation of aqueous humor behind the iris, further exacerbating the angle closure. Other RFs associated with PACG include female sex, older age, ethnicity, shallow anterior chamber depth, short axial length, and lens and iris dimensions [128].

Worldwide, PACG is less common than POAG [1], however in certain populations the prevalence of PACG is significantly higher than the prevalence of POAG. For example, PACG is considerably more prevalent in Asian populations, and populations of Asian descent such as Greenlandic and Alaskan Inuit populations than populations of ED, AD, and LAD [16,17,18,19,20,23,24,25,26,68,129]. In fact, more than 75% of global PACG cases are present in Asian populations [2,130]. This worldwide disparity has been hypothesized to be the result of several factors including thicker irises among these populations and [131], at least among Inuit populations, possibly an evolutionary thermoregulatory byproduct [21,129,132,133]. Regardless, these epidemiologic differences suggest the role of genetics in the underlying pathology of PACG.

#### 3.2.1. Asian Descent

The disease burden of PACG in populations of Asian descent is high. According to data from the World Health Organization in 2010, PACG was the reported cause of blindness in approximately 1.3 million people, and this number was expected to increase by 50% by 2020 [134]. Additionally, the CGSC noted PACG to be responsible for almost 80% of glaucoma cases in China [90]. In Asian populations, the heritability of PACG has been cited to be as high as 60–65% [135,136].

Similar to POAG, GWAS have become the best approach to studying the genetics of PACG given the ability to screen large numbers of SNPs in a large number of patients. Prior to GWAS studies, animal models—specifically canine and mouse models—were used to identify possible susceptibility loci [9], as well as studies looking at specific single-gene variants that could lead to increased susceptibility to PACG through ocular RFs such as extracellular matrix (ECM) remodeling and its effect on axial length and IOP [137,138]. These studies identified *MMP9* SNPs to be implicated in ECM remodeling, which can lead to shorter axial length as well as elevated IOP. The association between MMP SNPs and PACG has been confirmed and expanded in both Asian and ED populations [9,139,140].

In addition to *MMP*9, other single-gene variants that have been identified to be connected to PACG include *HGF* (hepatocyte growth factor), *HSP70* (heat-shock protein 70), *MFRP* (membrane type frizzled related protein), and *eNOS* (endothelial nitric oxide synthase) [126], though these studies require confirmation in different populations [126,141].

Some single-gene variants have been discovered that may impact RFs for PACG. For example, in 2014, Nongpiur et al. examined SNPs within *ABCC5* (ATP binding cassette subfamily C member 5) and its contribution to anterior chamber depth (ACD) through GWAS [142]. These findings were then expanded to find more SNPs in the genetic region associated with *ABCC5* in 2017 [143]. Khor, et al., however, were unable to find the association between *ABCC5* and PACG in their multi-ethnic analysis which suggested that ACD endophenotypes may not capture the complete risk [144].

In 2012, Vithana, et al. conducted a GWAS across Asia and identified three new PACG susceptibility loci in an Asian population [145], two of which (rs11024102 in *PLEKHA7*, and rs3753841 in *COL11A1*) were confirmed in a large PACG cohort study in China in 2014, and in a PACG cohort from Australia and Nepal in 2013 [146,147]. An intergenic locus, rs1015213, between *PCMTD1 and ST18* was similarly confirmed in a PACG cohort from a South Indian population in 2013 [148].

In 2016, the multi-ethnic GWAS from Khor, et al. identified five new genetic loci for PACG among 10,503 cases and 29.5667 controls. They identified SNPs in *CHAT* (OR 1.22, *p* = 2.85 × 10^−16^)*, DPM2*–*FAM102A* (OR 1.15, *p* = 8.32 × 10^−12^), *EPDR1* (OR 1.24, *p* = 5.94 × 10^−15^), *FERMT2* (OR 1.14, *p* = 3.43 × 10^−11^), and *GLIS3* (OR 1.18, *p* = 1.43 × 10^−14^), and confirmed the previously reported associations of SNPs at *PLEKHA7, COL11A1,* and *PCMTD1-ST18* (*p* < 5 × 10^−8^) [144,145].

Similar to POAG genetic studies, the next step from GWAS was PRS studies. In 2020, a case-control study of patients of Chinese ethnicity from Singapore confirmed the association to the 8 SNPs identified by Khor, et al. in 2016. They observed a significant association with severe PACG at rs3816415 in *EPDR1* (OR 2.03, 95%CI: 1.49–2.78, *p* = 1 × 10^−5^) suggesting that patients with this genetic variant may be predisposed to an aggressive form of PACG. Additionally, they found that a higher PRS was associated with a higher risk of severe PACG (OR 3.11, 95%CI: 1.95–4.96) [149]. Interestingly, in 2019 the same team of researchers used these 8 SNPs to calculate a PRS and, in a case-control study, found that the inclusion of risk alleles (either alone, or as a PRS) to other traditional diagnostic criteria (specifically anterior chamber depth) only resulted in a +0.50% improvement in diagnosis of PACG cases from the baseline discriminatory value of ACD, and this improvement was not statistically significant (*p* > 0.05) [150].

Alternatively, in 2019, a GWAS was performed based on single-gene variants that had been previously identified via-WES data. Initial WES was performed on 549 samples and was compared to WES data from 2747 probands of other inherited eye diseases. Researchers identified 723 genes with potential pathogenic polymorphisms, and then confirmed these genes in a WES dataset from 4327 East Asians. The analysis identified five novel genes: *ACTBL2* (*p* = 0.04), *BEX1* (*p* = 2.0 × 10^−4^), *DNMT3A* (*p* = 1.1 × 10^−3^), *LDLRAD2* (*p* = 2.2 × 10^−3^), and *SIN3A* (*p* = 7.0 × 10^−4^). Additionally, seven genes known to be associated with other inherited eye diseases showed significantly enriched coding variants (ECVs): *BEST1* (*p* = 0.04), *FBN1* (*p* = 0.01), *FOXC2* (*p* = 0.01), *HK1* (*p* = 4.0 × 10^−4^), *OPTN* (*p* = 7.7 × 10^−3^), *PIEZO2* (*p* = 7.3 × 10^−4^), and *TTR* (*p* = 6.7 × 10^−3^) (Table 2) [151]. The authors, however, suggested that addition study was required to confirm these findings, and PRS may be a way to further expand these associations.

#### 3.2.2. European Descent

As opposed to Asian populations, the prevalence of PACG is low in Europe (0.42%) and North America (0.26%) [25,126], and genetic data on PACG in ED is generally lacking [126]. The low prevalence makes study and identification of affected ED families difficult [156].

In an Australian Caucasian population, SNPs in the MMP-9 gene have been associated with PACG but have not been replicated [140]. Similarly, in a population from Quebec, mutations in MYOC have been associated with glaucoma, but these reports have not been consistent when applied to other populations [127,157,158]. 

In 2012, Awadalla, et al. examined PACG among a cohort of Nepalese and Australian patients and matched controls. They found an association between SNPs in *MFRP* to be associated with both cohorts, though they were not associated after they were adjusted for sex and age. Meanwhile, they found an association between an SNP of the *CALCRL* gene in the Australian population (*p* = 0.024), but no association for either population and SNPs in *MTHFR* [153].

More recently, in 2020, Waseem, et al. conducted a study of PACG in an ED family in the UK [156]. They studied 39 blood-related patients, 5 of which were unaffected (penetrance 87.2%). This study identified SNPs of the *SPATA13* gene as the causal gene for PACG in this family. Specifically a 9 base pair deletion was implicated in all of the patients who had PACG. When *SPATA13* was then studied in a cohort of 189 unrelated individuals, the researchers found eight additional mutations associated with PACG. Importantly, this was the first study to identify *SPATA13* as a gene that was associated with both PACG and any eye disease, though GWAS have implicated the gene product (protein SP-1277) with other disorders such as anorexia nervosa, thyroid cancer, and intellectual disability [156].

#### 3.2.3. Middle Eastern Descent

Across the ME there have been different reports of the prevalence of PACG. For example, in 2005 the Oman Eye Study reported the prevalence of PACG at 0.02% [159]. Meanwhile, in Saudi Arabia, PACG has been reported as the primary type of adult glaucoma (46.6% compared to 25.6% POAG) [160], and several studies have noted that PACG in Saudi Arabia is closer to estimated prevalence for Asian populations [25,127]. Additionally, familiarity of PACG has been confirmed in a 2015 Iranian study which showed that 2/3 of siblings with PACG had clinical findings related to angle closure [161], a finding that reflects heritability trends in Asian populations [135,136]. 

In 2020, a study in Northeast Iran aimed to evaluate the five polymorphisms that had been previously identified by the 2016 multi-ethnic GWAS from Khor, et al. The researchers identified significant associations between PACG and the SNPs for *CHAT* (*p* = 0.02), *EPDR1* (*p* < 0.001), *FERMT2* (*p* < 0.001), and *GLIS3* (*p* = 0.005), but not *DPM2-FAM102A* [154].

A variety of other single-gene variants have been associated with PACG in ME populations. For example, Pakistani populations have reported polymorphisms of *eNOS* and *HSP70* to be associated with PACG, as well as *MMP9* [155]. The eNOS polymorphism was also found to be associated with POAG [58]. In 2017, a case-control study associated the GA genotype of the *SMOC2* (G > A) polymorphism with PACG in a SA population [162]. Additionally, in 2020 a SNP in the *ACVR1* gene involved in the bone morphogenic protein (BMP) signaling pathway was associated with both PACG and PXG in a SA cohort [152].

Several studies have also identified polymorphisms that were not significantly associated with PACG in a SA population, but rather with clinical indices for PACG, suggesting that these SNPs could possibly be used as indicators for PACG severity. In 2013, *SOD2* polymorphisms were associated with mean age of PACG onset, duration of onset, and mean visual acuity [163], while a CAT promoter polymorphism has similarly been associated with visual acuity [164].

Similar to POAG, GSTM1 null polymorphism has been studied for association with PACG in ME patients. In 2015, it was determined that there were increased frequencies of GSTM1 null in Iranian patients with PCAG, leading the authors to suggest that GSTM1 null could be associated with a risk factor for PCAG incidence in Iranian populations [165]. In fact, a possible association between GSTs and glaucoma in Arab patients has been cited as far back as 2007 [166].

Finally, an interesting study from 2018 associated *COL18A1,* which encodes collagen type XVIII, as a causative gene for angle closure in a pedigree with at least 10 individuals with PAC. *COL18A1*, however, has been associated with Knobloch syndrome, which itself is characterized by severe vision problems including high myopia and retinal detachment. *COL18A1* was identified in two other cases of PACG in unrelated families, but these two individuals were parents and grandparents of KS-affected children [167]. 

#### 3.2.4. Latin American Descent and African Descent

The prevalence of PACG in LAD and Caribbean populations is low (0.85%), but is still higher than the prevalence of PACG in ED populations [24,126]. Similarly, the prevalence of PACG is low in AD populations, with studies citing prevalence anywhere from 0.2% to 0.6% [26,126,168]. As a result, there are very few studies that have examined the genetic connections between PACG and the LAD or AD populations.

The 2016 multi-ethnic GWAS from Khor, et al. is the only study to demonstrate a genetic association for LAD populations, as the studied population included cases and controls from South American countries in the analysis [144]. Meanwhile, one study on elderly African American males in Brazil reports that plateau iris configuration (PIC), a noted RF for PACG, may be associated with long anterior zonules (LAZ) and, therefore, LAZ may be a useful clinical indicator for an increased risk of PIC and, therefore, PACG. This study notes, however, that the mutation that is classically associated with LAZ, *CTRP5* S163R, were not present in the studied patients [169].

Ultimately, given the difficulties of replicating GWAS and PRS across ethnicities [34,36], further studies are needed to address PACG in LAD and AD populations. Next steps may include performing WES in PACG-impacted families (similar to Waseem, et al. in the UK [156]), or gathering large databases of glaucoma patients across the regions to prepare for GWAS, or even single-gene analysis, in such populations where PACG prevalence is low. 

### 3.3. Exfoliation (Pseudoexfoliation) Glaucoma

XFG is differentiated from POAG and PACG in that XFG occurs as a part of exfoliation (pseudoexfoliation) syndrome (XFS), which is an age-related disorder characterized by systemic deposition of fibrillar extracellular material throughout the body. In the eye, these depositions commonly occur in the anterior segment, leading to a form of open-angle glaucoma that typically progresses more rapidly than POAG and has significantly worse visual field damage and a higher severity of optic nerve damage [170,171,172]. Interestingly, the prevalence of XFS/XFG is widely varied among populations and even within different ethnic groups, strengthening the argument for a genetic explanation for XFG.

The basis for most genetic study of XFS/XFG is a 2007 GWAS from Thorleifsson et al., which found an association between XFS/XFG and three SNPs of the *LOXL1* (lysyl oxidase-like 1) gene on chromosome 15q24 (Table 3) [173]. Study of these SNPs, rs1048661, rs3825942, and rs2165241, has since been repeated in various populations and expanded. Importantly, several studies of these SNPs have demonstrated that they do not appear to be associated with the pathogenesis of POAG—a key distinction since XFS/XFG is a cause of secondary open-angle glaucoma [174,175,176]. Furthermore, it has been suggested that disease is the result of build-up of *LOXL1* protein in the setting of decreased cellular proteostasis capability due to aging [177], and *LOXL1* has also been implicated in IOP changes, as decreased expression results in changes to ocular outflow physiology [178].

In 2015, a second locus, *CACNA1A*, was identified to be associated with XFS/XFG. A GWAS of 1484 patients and 1188 controls from Japan, and then confirmed on a global sample of 6901 patients and 20,727 controls discovered a significant association between *CACNA1A* rs4926244 and increased risk of XFS/XFG (OR 1.16, *p* = 3.36 × 10^−11^). Additionally, this study confirmed the association between *LOXL1* and XFS/XFG. This study was significant as it identified the first genetic locus outside of *LOXL1* to be significantly associated with XFS/XFG [181].

Genetic associations with XFS/XFG, however, are expanding beyond *LOXL1* and *CACNA1A*. In 2017, a GWAS using a global sample of 5570 XFS/XFG cases and 6279 controls identified five new variants: *POMP, TMEM136, AGPAT1, RBMS3,* and *SEMA6A*. This study also demonstrated that a rare LOXL1 missense variant may be protective against XFS/XFG [179]. 

Aside from genetics, it’s important to note that the widely varied prevalence of XFS/XFG among different population groups could also strengthen an argument for varied environmental exposures. In fact, geo-medical studies have shown that disease burden seems to increase as populations are more distant from the equator [27]. The key environmental factor in this hypothesis is ocular UV exposure, which is difficult to study given the lack of an accepted biomarker of UV exposure–so researchers generally correlate disease with time spent outdoors. Researchers have tried to tie in environmental factors with genetics to gain a better understanding of XFS/XFG RF. Generally, hypotheses suggest that the environment and genetics work in concert, where environmental exposures induce susceptibility by weakening the blood-eye-barrier in the eye, leading ECM proteins in the anterior chamber to get cross-linked by *LOXL1* protein expression leading to disease [27]. Still, these hypotheses require further research both taken together and individually and in different populations. For example, XFS/XFG has not been detected in Greenlandic Inuit populations, but this can be attributed to narrow lid fissures and thick irises which protect them from ocular UV radiation and, therefore, an environmental-genetic cascade of disease [10,11,189].

#### 3.3.1. European Descent

XFS is most common in Greek and Nordic populations, with a prevalence as high as 10% in Iceland and 20% in Swedish people over the age of 60 [190,191]. In the Greek population, specifically Thessaloniki, longitudinal follow-up demonstrated XFG to have a 12-year incidence of 19.6% (95%CI: 17.1–22.2), with women more likely to be affected than men (*p* = 0.0197), indicating a large disease burden in this population [192].

The 2007 study from Thorleifsson, et al., which established loci in *LOXL1* as genetic RFs for XFS/XFG, was performed in a Caucasian population [173]. In most populations of ED, including Swedish/Icelandic, US Caucasian, Australian, and European populations, rs3825942 (Gly153Asp) is strongly associated with risk for XFS/XFG, as well as for all other populations studied except for South Africans, where the risk allele is reversed [171]. Meanwhile, rs1048661 has not been associated with XFG risk in Greek and Polish populations [11]. In a US Caucasian population, *LOXL1* promoter region SNPs have also been associated with XFS/XFG [193].

Importantly, ED populations are perhaps the strongest populations to demonstrate the possible importance of environmental factors of XFS/XFG. In the United States, several studies have demonstrated that individuals who live in northern regions of the country had an increased risk of XFS when compared to individuals living in middle and southern regions of the country. Generally, these studies found that risk of XFS corresponded with the average number of sunny days, indicating a possible role for altitude and UV exposure to pathogenesis of XFS and XFG [194,195,196]. These studies are intriguing given that the *LOXL1* association is still present in patients with XFS from the Midwest United States [197]. Additionally, though not of ED, prevalence rates up to almost 40% have been reported in Navajo Native American populations, likely given time spent outdoors and UV light exposure [11].

These findings about the possible role of environmental factors are supported by genetic characteristics described in an Australian Caucasian population. In 2007, Hewitt, et al. noted that the lifetime incidence of XFS in Caucasian Australians was nine times lower than that of Nordic populations, despite the *LOXL1* locus having similar genetic architecture in both populations [198].

Meanwhile, in 2010, the Reykjavik Eye Study found XFS also correlated with increased iris pigmentation, and noted a possible antioxidant protective effect, suggesting a possible oxidative stress mechanism for XFS [191]. 

#### 3.3.2. African Descent

In populations of AD, the prevalence and distribution of XFS/XFG is widely varied. XFS is rare in African Americans [199,200], but has a strange distribution throughout the African continent. In West African countries such as Ghana, XFS is basically not reported [201,202]. In black South Africans, by contrast, XFG is the reported cause of almost 20% of glaucoma cases [203,204]. In East Africa, similarly, a facility-based cross-sectional study determined that XFG was the most common subtype of glaucoma at 35.2% (POAG 32.8%), but these results have not been reported elsewhere [205], and a separate Ethiopian study showed XFG to be 26.6% of glaucoma cases with 37.7% attributed to POAG [206]. Regardless, the disease burden of XFG appears to be high in select populations of AD, possibly due to climate and UV exposure [168,170,199,200,201,202,203,204,205,206].

In 2010, Williams et al. conducted the first study of *LOXL1* in an ancestral African population with XFG, specifically a black South African population. They confirmed the association between *LOXL1* SNPS, specifically rs1048661 and rs3825942, and XFG [171]. Additionally, they found the risk allele for rs3825942 was the A allele, rather than the G allele previously described in non-African populations [171]. This finding was later confirmed in a 2011 case-control study showing that the AA genotype of G153D (rs3825942) was associated with XFS/XFG risk in this population rather than the GG genotype [207].

In 2015, Hauser et al. determined a functional role for the *LOXL1* variants associated with XFS/XFG in South African XFS cases. They demonstrated that the relevant SNPs impacted a region containing a promoter and, therefore, disrupt the activity of the promoter. However, the identified region does not lead to increased *LOXL1* promoter activity, so the pathogenetic role of these SNPs is unclear. They suggest that altered expression of *LOXL1*-*AS1*, the long non-coding RNA, leads to XFS pathogenesis via-dysregulated cellular stress response [208]. These findings were replicated in Caucasian, German, and Japanese populations [208]. However, it is important to note that many gene variants have different functions not related to disease pathogenesis, and these findings also do not consider environmental exposures.

#### 3.3.3. Asian Descent

A 2015 retrospective chart review of 73,946 inpatients at a Beijing hospital showed only 45 patients (75 eyes) identified, suggesting that XFS/XFG is uncommon in this specific urban Chinese population [209]. Similarly, the CGSC, the first nationwide glaucoma registry in China, reported only 41 cases of XFG in a population of 5762 glaucoma cases (0.71%) [90]. Meanwhile, in the Novosibirsk region of Russia, the XFG appears to account for 70% of open-angle glaucoma cases, much higher than the corresponding rates in European-Russia, suggesting possible environmental influences [210]. 

Despite the low prevalence of XFS/XFG, studies have shown that *LOXL1* polymorphisms confer similar risk of XFS/XFG in Chinese populations [211,212,213]. Given the similar risk in the setting of lower incidence, authors suggested that XFS may have other genetic or environmental factors that influence phenotypic expression of XFS/XFG in this Chinese population. Interestingly, though, Uygur populations in China have higher rates of XFS/XFG—with rates as ranging between 2.2% to 9.5% depending on age [214]. In these populations, *LOXL1* SNPs have similarly been identified as risk alleles for XFS/XFG [180,215]. Uygur populations have also shown association with SNPs of *TBC1D21* and *ATXN2* [216]. Meanwhile, in North Indian populations, rs3925942 has shown a significant association with XFS/XFG [217], but other SNPs of *LOXL1* have shown no association [182].

In 2017, Pasutto, et al. performed a GWAS on 771 German XFS/XFG patients and then independently tested the associated gene variants in Italian and Japanese data sets [218]. The researchers sought to connect the established *LOXL1* genetic findings to a possible mechanism and explain variation in the *LOXL1* expression phenotype. They argued that increased transcription of the risk sequence results in elevated levels of an irregularly spliced *LOXL1* transcript leading to decreased levels of normal *LOXL1* mRNA. They identified the transcription factor RXRalpha, and regulated coupling of alternative splicing and nonsense-mediated decay, as the keys to the mechanism of *LOXL1* gene regulation [218]. 

Importantly, Pasutto, et al. did note as a limitation of the study, that the XFS/XFG risk alleles were identical in the German and Italian populations, but reversed in Japanese populations, with matches other studies in Asian populations compared to Caucasian studies [212,219,220,221,222,223]. Additionally, they suggested several possible reasons for allelic reversal in these populations, including allelic heterogenicity, multi-locus effects, and the possibility of other underlying genetic and environmental RFs that result in observed phenotypes [218].

Meanwhile, in Japan, a 2014 GWAS further explored the inversion of the risk allele in *LOXL1* variants and found 34 SNPs in *LOXL1* but also *TBC1D21* and *PML*, both also located on chromosome 15q24.1. Upon further analysis, they found a suggestive combinational effect between *LOXL1* and *TBC1D21* which seemed to be specific to Asian populations, and found only one *TBC1D21* SNP to have a strong association while the *PML* SNPs were weaker [183]. Additionally, Japanese studies have demonstrated a role for SNPs of the *TLR4* gene in the pathogenesis of both POAG and XFG, but noted a strong association with NTG [224].

#### 3.3.4. Middle Eastern Descent

Prevalence of XFS/XFG in the Middle East is similarly varied. A retrospective chart review from Egypt in 2011 showed a 4.14% prevalence in 7738 patients who attended a general ophthalmology clinic. This indicates that XFG is relatively common in Upper Egypt [225]. Similarly, a cross-sectional population-based Turkish prevalence study demonstrated an XFS prevalence of 5.7% [226], while a prospective Pakistani prevalence study showed an XFS prevalence of 6.45% [227].

Similar to other populations, study of XFS in ME populations has focused on *LOXL1* gene polymorphisms. In 2010, a Saudi Arabian study demonstrated similar findings to other non-African populations, showing associations between the G allele of both rs1048661 and rs3825942 SNPs with XFG [184]. Several studies in other ME populations, including Turkish and Pakistani patients, have confirmed these associations [228,229].

In a 2017 study from Turkey, however, researches demonstrated that rs1048661 of *LOXL1* was not significantly associated with XFS/XFG in the Turkish population [185]. Additionally, they also reported the risk allele of rs3825942 to be the A allele, similar to African populations and opposed to other results finding the G allele as the risk allele, and reported a new association of rs2165244 [185]. Meanwhile, a 2016 analysis in Turkish patients with XFG showed that *LOXL1* SNPs were only present in 35.3% of patients. Additionally, they demonstrated that the patients with the *LOXL1* gene variants had no statistically significant differences in RNFL thickness and cup-disc ratio compared to patients without the SNPs, suggesting that *LOXL1* mutations may not play a role in XFS/XFG severity [230]. These conflicting findings actually support the hypothesis that *LOXL1* is not a gene for XFS and is rather a marker of the disease process that results in XFG.

Interestingly, Middle Eastern studies have also looked at other genes that may be associated with XFS/XFG. Similar to both POAG and PACG, GSTM gene polymorphisms have been studied with a variety of results. A 2010 study showed an association between GSTT1 and GTM1 genotypes with XFG in a cohort of female Pakistani patients [231]. Additionally, a 2005 Turkish study showed no association between GSTM1, GSTP1, and GSTT1 gene polymorphism in XFS [232]. Additionally, in Iranian populations, IL-10 gene promoter polymorphisms were shown to be associated with susceptibility to XFS/XFG and POAG [93]. More recently, in 2020, a SNP in the *ACVR1* gene involved in the BMP signaling pathway was associated with both PACG and PXG in a Saudi cohort [152]. This was the first study to associate this variant with PACG and PXG.

#### 3.3.5. Latin American Descent 

Little is known about the prevalence of XFS/XFG in LAD populations [233]. A 2007 study from Argentina reports that XFS/XFG could be high in an elderly Argentinian population, but this study was small and limited [234].

In 2012, Jaimes, et al. conducted the first study associating *LOXL1* polymorphisms with XFS/XFG in LAD populations. They performed a case-control study of a Mexican population and noted an elevated risk of XFS/XFG in this population when affected by *LOXL1* variants [187]. Another *LOXL1* SNP was identified in this population, the T allele of SNP rs41435250, in 2013 [186].

### 3.4. Key Pathways and Limitations for Future Research

Given the complex nature of glaucoma, and its non-Mendelian inheritance pattern, researchers have tried to further characterize glaucoma-associated genes using large-scale agnostic searches among mostly unrelated cases and controls that incorporate complex computational tools. In 2017, Danford et al. conducted a bioinformatics-based review of the “POAGome” (including phenotypes not included in this analysis such as juvenile open-angle glaucoma and primary congenital glaucoma). Using DisGeNET, an Integrative Biomedical Informatics Group database, they collected a list of 542 associated genes, and reviewed and analyzed possible pathways for disease development based on both specific ocular tissues and different phenotypes of disease. Ultimately, they suggested that there was no unified molecular pathway that could be the single responsible mechanism for POAG pathogenesis while noting that the inflammation and senescence in the TGF-β signaling pathway may play an important role in glaucoma development [188]. Undoubtedly there are additional pathways involved and significantly more data on differing populations, is required to understand whether or not there are common genetic RFs and, in turn, common pathways for disease pathogenesis and progression.

Meanwhile, it is similarly important to understand that the calculation of PRS presents several limitations that have yet to be overcome. For one, the actual application of risk scoring to broad-scale clinical use is still not widespread, indicating that the relevance is currently more abstract and requires work to reach the point of clinical implementation. Additionally, it’s important to again note the challenges associated with applying PRS across ethnic groups. According to Lewis and Vassos, current PRS analysis relies largely on the assumption that an individual’s genetic ancestry reflects the large GWAS where scores are calculated from—an issue compounded by the fact that the majority of these studies globally are reliant on ED populations [235]. Importantly, efforts to create “polyethnic” scores are being developed [236,237]. Additionally, although PRS may show significant associations with disease—as many cited in this review do—the utility of these scores is difficult to understand. For example, it is unclear whether or not these scores are able to be used for risk stratification or disease prediction, and it is even more unclear how clinicians and patients should react to these scores. 

In addition to the utility of PRS, there are several challenges in computing them. For example, according to Igo Jr. et al., the complexities of glaucoma and the definitions of its varying phenotypes present a challenge when determining the outcome of interest in the PRS. For example, in POAG, PRS may be calculated based on endophenotypes, IOP, VF loss, or other parameters alone or together [238]. From there come challenges of interpreting scores. According to Igo Jr. et al., predictive models utilizing PRS are usually assed with measure of AUC, which generally ranges from 0.5 (even chance) to 1 (perfect model), with an expectation of AUC > 0.75 for informative screening [238]. For example, the three PRS calculated in the ADAGES III analysis had AUC values of 0.62, 0.74, and 0.94, suggesting varying utility among them [29]. Other calculations used to demonstrate the strength of PRS are more traditional statistical analyses. Given the complexities of calculating and analyzing these scores, statistical analysis software packages have been written to assist researchers [238].

Artificial intelligence and mathematical modeling approaches, alongside deeper genotype-phenotype associations, may provide greater clarity of underlying RFs and mechanistic pathways for disease. For example, Guidoboni et al., have created mathematical models for ocular blood flow in order to understand underlying vascular RFs [239]. Similarly, Seo and Cho utilized deep learning techniques, specifically a deep neural network, to evaluate the association between specific optical coherence tomography-based parameters and NTG [240]. As these techniques develop further and more genetic information is made available for use, these research techniques hold promise in elucidating a genetically driven model for glaucoma pathogenesis. Additionally, the confluence of predictive modeling and traditional statistical analysis with artificial intelligence technology and other computational tools holds the promise of dramatically influencing the clinical practice of glaucoma [241]. 

As for PACG and XFG, there is currently a lack of mathematical modeling and artificial intelligence applications for these disease presentations—perhaps given factors such as disease burden, population characteristics, and better-understood pathophysiologic properties. Particularly in the case of XFG, there seems to be a general consensus on the involvement of a limited number of genes, pointing to a mechanism involving *LOXL1* expression [10]. 

Finally, it’s important to note that the practice of genetic and genomic analysis is still evolving, with a variety of challenges to analysis including bioinformatics expertise. In 2019, Jeong et al. published a new web-based algorithm, called CRISPRBetaBinomial, to overcome these challenges and to make CRISPR analysis more easily accessible to researchers in addition to having greater sensitivity and fewer false negatives [242]. While this technology has not yet been applied to the study of glaucoma genes, it is an example of the future of genetic analysis and the promise it holds in the study of glaucoma and other ocular diseases.

## 4. Conclusions

Glaucoma is the worldwide leading cause of blindness, with increasing prevalence and a disease burden that varies by glaucoma subtype and ethnicity. Genetic and genomic studies have demonstrated the potential for genetic RFs in glaucoma; however, several challenges remain before their clinical application is realized.

Generally, there remains a resource-allocation and distribution challenge that mirrors global development. Populations with better access to technologic advances, specifically ED and Asian populations, are largely progressing past the assessment of single-gene variants into GWAS, which allow for the creation of more detailed PRS and, possibly, a clinical tool. Genetic studies, however, have proven to have difficulty crossing ethnicities, a challenge demonstrated by POAG in populations of AD. AD populations are disproportionately affected by POAG and yet there is not substantial research on the genetics of these populations. This creates a knowledge gap that furthers the divide between disease burden and the resource allocation of the most affected populations.

Meanwhile, other studies are challenged by the heterogeneity of populations. This is specifically true in the Middle East—where European, Asian, and African ancestry, along with consanguinity, muddies the water of analysis—and in Latin America, where a similar melting pot of ancestry seems to create a diversified genetic population. Additionally, genetic disparities between and within each population have made discernment of a common genetic pathway of glaucoma pathogenesis difficult. Therefore, genetic studies exploring endophenotypes within individual ethnic groups may elucidate common mechanistic pathways. 

Undoubtedly, a future direction of study is to understand the genetic basis for disease burden in these populations. Genetic and genomic research has shown that common gene variants are RFs for POAG and have shown that IOP and cup-disc ratio are important endophenotypes for POAG, so PRSs show promise for clinical impact in these populations once genetic data is available. As for PACG, questions remain on what relevant endophenotypes may be and, given the disappointing state of PACG PRSs, much more genetic study is warranted to elucidate a genetic basis for disease. Meanwhile, further study is needed to determine if XFG is even a genetic disease or rather an environmental effect that is modulated by gene expression. It is also important to note alleles may flip in different populations and as described above *LOXL1* does not track disease burden so much as its expression parallels environmental exposures. 

In order for genetic analysis to expand and become useful tools in the clinic among these populations, large scale carefully designed studies are required; perhaps ones similar to the model of the UK Biobank or similar large long-term study. As more genetic data becomes available, the expansion of GWAS and PRS may eventually allow for the ability for earlier glaucoma detection and, possibly, genetic-based therapeutics. The application of artificial intelligence on determining individualized risk from glaucoma RFs that include genetic considerations may also further improve specificity of patient care.

## Figures and Tables

**Table 1:** Genes associated with primary open-angle glaucoma and normal tension glaucoma. SNP: single nucleotide polymorphism.

Gene	SNP/Genotype	Protein Product	Gene Function	Ethnicity	Reference
*ABCA1*	rs2472493	ATP Binding Cassette Subfamily A Member 1	Molecular transport	European Descent	Gharahkhani et al., 2014 [37]
*ABCA1*	rs2487032	ATP Binding Cassette Subfamily A Member 1	Molecular transport	Asian Descent	Chen et al., 2014 [43]
*ABCA1*	rs2472493	ATP Binding Cassette Subfamily A Member 1	Molecular transport	Asian Descent	Hysi et al., 2014 [44]
*ABCA1*	rs2472493	ATP Binding Cassette Subfamily A Member 1	Molecular transport	Multi-Ethnic	Choquet et al., 2018 [45]
*ADAMTS8*	rs56009602	ADAM Metallopeptidase with Thrombospondin Type 1 Motif 8	Protein metabolism	Asian Descent	Iglesias et al., 2018 [46]
*AFAP1*	rs4619890	Actin Filament Associated Protein 1	Cross-linking actin filaments	European Descent	Gharahkhani et al., 2014 [37]
*AFAP1*	rs4619890	Actin Filament Associated Protein 1	Cross-linking actin filaments	Asian Descent	Shiga et al., 2018 [47]
*AFAP1*	rs59521811	Actin Filament Associated Protein 1	Cross-linking actin filaments	Multi-Ethnic	Choquet et al., 2018 [45]
*ANKH*	rs76325372	ANKH Inorganic Pyrophosphate Transport Regulator	Mediates control of pyrophosphate levels	Multi-Ethnic	Choquet et al., 2018 [45]
*ANKRD55-MAP3K1*	rs61275591	Ankyrin repeat domain-55-Mitogen-activated protein kinase kinase kinase 1	Expressed in ocular tissues	Asian Descent	Shiga et al., 2018 [47]
*APBB2*	rs59892895	Amyloid-beta A4 precursor protein-binding family B member 2	APP processing in retina and primary visual cortex	Middle Eastern	Hauser et al., 2018 [48]
*ARHGEF12* ^1^	rs58073046	Rho Guanine Nucleotide Exchange Factor 12	RhoA/RhoA kinase pathway and IOP regulation	European Descent	Springelkamp et al., 2015 [42]
*ATXN2*	rs7137828	Ataxin 2	Ataxin 2 production within cell cytoplasm	European Descent	Bailey et al., 2016 [38]
*C12ORF23*	rs1333037	Transmembrane protein C12orf23	Associated with growth and bone development	European Descent	Bailey et al., 2016 [38]
*CADM2*	rs34201102	Cell Adhesion Molecule 2	Regulated trans-synaptic cell adhesion	Multi-Ethnic	Choquet et al., 2018 [45]
*CAV1*	rs4236601	Caveolin 1	Expressed in eye development	European Descent	Thorleifsson et al., 2011 [39]
*CAV1*	rs10258482	Caveolin 1	Expressed in eye development	Asian Descent	Hysi et al., 2014 [44]
*CAV1-CAV2*	rs4236601	Caveolin 1	Expressed in eye development	Asian Descent	Lu et al., 2020 [49]
*CDKN1A*	rs6913530	Cyclin Dependent Kinase Inhibitor 1A	Regulates cell cycle progression	Multi-Ethnic	Choquet et al., 2018 [45]
*CDKN2A-CDKN2B*	rs1063192	Cyclin-dependent kinase 4 inhibitor B	Tumor suppressor genes	Asian Descent	Osman et al., 2012 [50]
*CDKN2B-AS1* ^1^	rs2157719	Non-protein coding	Regulates CDKN2A and CDKN2B	European Descent	Wiggs et al., 2012 [51]
*CDKN2B-AS1* ^1^	rs4977756	Non-protein coding	Regulates CDKN2A and CDKN2B	European Descent	Burdon et al., 2017 [52]
*CDKN2B-AS1* ^1^	rs1333037	Non-protein coding	Regulates CDKN2A and CDKN2B	European Descent	Bailey et al., 2016 [38]
*CDKN2B-AS1*	rs79721419	Non-protein coding	Regulates CDKN2A and CDKN2B	African Descent	Taylor et al., 2019 [29]
*CDKN2B-AS1*	rs10712703	Non-protein coding	Regulates CDKN2A and CDKN2B	African Descent	Bonnemaijer et al., 2018 [53]
*CDKN2B-AS1* ^1^	rs523096	Non-protein-coding	Regulates CDKN2A and CDKN2B	Asian Descent	Takamoto et al., 2012 [54]
*CDKN2B-AS1*	rs4977756	Non-protein coding	Regulates CDKN2A and CDKN2B	Asian Descent	Shiga et al., 2018 [47]
*CDKN2B-AS1*	rs944800	Non-protein coding	Regulates CDKN2A and CDKN2B	Asian Descent	Shiga et al., 2018 [47]
*CDKN2B-AS1*	rs2157719	Non-protein coding	Regulates CDKN2A and CDKN2B	Latin American	Nunes et al., 2018 [55]
*CDKN2B-AS1*	rs10811645	Non-protein coding	Regulates CDKN2A and CDKN2B	Multi-Ethnic	Choquet et al., 2018 [45]
*DGKG*	rs9853115	Diacylglycerol Kinase Gamma	Enzyme for lipid metabolism	Multi-Ethnic	Choquet et al., 2018 [45]
*ELOVL5* ^1^	rs735860	Elongation of very long chain fatty acids protein 5	Enzymatic function	Asian Descent	Meguro et al., 2010 [56]
*EN04*	rs185815146	Enolase 4	Glucose metabolism	African Descent	Taylor et al., 2019 [29]
*eNOS/NOS3*	T-786C	Nitric oxide synthase 3	NO production	Latin American	da Silva et al., 2012 [57]
*eNOS/NOS3*	Glu298Asp	Nitric oxide synthase 3	NO production	Latin American	da Silva et al., 2012 [57]
*eNOS/NOS3*	intron 4 VNTR repeat	Nitric oxide synthase 3	NO production	Middle Eastern	Ayub et al., 2010 [58]
*EXOC2*	rs2073006	Exocyst Complex Component 2	Exocytic vesicle targeting	Multi-Ethnic	Choquet et al., 2018 [45]
*EXOC4*	rs141186647	Exocyst Complex Component 4	Exocytic vesicle targeting	African Descent	Bonnemaijer et al., 2018 [53]
*FMNL2*	rs56117902	Formin Like 2	Elongation of actin filaments	Multi-ethnic	Choquet et al., 2018 [45]
*FNDC3B*	rs111698934	Fibronectin Type III Domain Containing 3B	Regulates adipogenesis	African Descent	Taylor et al., 2019 [29]
*FNDC3B*	rs7636836	Fibronectin Type III Domain Containing 3B	Regulates adipogenesis	Asian Descent	Shiga et al., 2018 [47]
*FOXC1*	rs2745572	Forkhead Box C1	Transcription factor	European Descent	Bailey et al., 2016 [38]
*GAS7*	rs9897123	Growth arrest-specific protein 7	Neuronal development	European Descent	Bailey et al., 2016 [38]
*GAS7*	rs8080535	Growth arrest-specific protein 7	Neuronal development	African Descent	Taylor et al., 2019 [29]
*GAS7*	rs9913911	Growth arrest-specific protein 7	Neuronal development	Asian Descent	Hysi et al., 2014 [44]
*GMDS*	rs11969985	GDP-mannose 4,6 dehydratase	Protein modification and metabolism	European Descent	Gharahkhani et al., 2014 [37]
*HK2* ^1^	Rs678350	Hexokinase 2	Intracellular glucose metabolism	Asian Descent	Shiga et al., 2018 [47]
*HMGA2*	rs343093	High-mobility group AT-hook 2	Transcription factor	Asian Descent	Shiga et al., 2018 [47]
*IKZF2*	rs56335522	IKAROS Family Zinc Finger 2	Lymphocyte development	Mutli-Ethnic	Choquet et al., 2018 [45]
*IL1β*	–31C/T	Interleukin 1 Beta	Pyrogenic activity	Latin American	Oliveira et al., 2018 [59]
*IL1β*	–511C/T	Interleukin 1 Beta	Pyrogenic activity	Latin American	Oliveira et al., 2018 [59]
*LMX1β*	rs10819187	LIM Homeobox Transcription Factor 1 Beta	Transcription factor	Asian Descent	Shiga et al., 2018 [47]
*LMX1β*	rs55770306	LIM Homeobox Transcription Factor 1 Beta	Transcription factor	Multi-Ethnic	Choquet et al., 2018 [45]
*LOXL1*	rs1048661	Lysyl Oxidase Like 1	Connective tissue biogenesis	Asian Descent	Shiga et al., 2018 [47]
*LRP12/ZFPM2*	rs284491	LDL Receptor Related Protein 12/Zinc Finger Protein	Endocytosis and neuron migration/transcriptional activation, regulation of apoptosis, lipid binding	European Descent	Bailey et al., 2016 [38]
*MEIS2*	rs28480457	Meis Homeobox 2	Transcription factor	Asian Descent	Shiga et al., 2018 [47]
*MMP9* ^1^	rs2274755	Matrix metalloproteinase 9	Regulates pathological remodeling processes	Asian Descent	Suh et al., 2018 [60]
*NCK2* ^1^	rs2033008	NCK Adaptor Protein 2	Regulates synaptic transmission	Asian Descent	Shi et al., 2011 [61]
*PDE7B*	rs9494457	Phosphodiesterase 7B	Downregulates cAMP and cGMP signaling	Latin American	Choquet et al., 2018 [45]
*PLXDC2*	rs7081455	Plexin Domain Containing 2	Cell surface binding to PEDF	Asian Descent	Nakano et al., 2009 [62]
*PMM2*	rs3785176	Phosphomannomutase 2	Glycosylation enzyme	Asian Descent	Chen et al., 2014 [43]
*SIX1/SIX6* ^1^	rs10483727	Homeobox protein SIX1-SIX6	Transcription factors	European Descent	Bailey et al., 2016 [38]
*SIX1/SIX6* ^1^	rs33912345	Homeobox protein SIX1-SIX6	Transcription factors	European Descent	Wiggs et al., 2012 [41]
*SIX1/SIX6*	rs10483727	Homeobox protein SIX1-SIX6	Transcription factors	Middle Eastern	Kondkar et al., 2018 [63]
*SIX1/SIX6*	rs35155027	Homeobox protein SIX1-SIX6	Transcription factors	Multi-Ethnic	Choquet et al., 2018 [45]
*SIX6*	rs10483727	Homeobox protein SIX6	Transcription factor	Asian Descent	Shiga et al., 2018 [47]
*SBRD1* ^1^	rs3213787	S1 RNA Binding Domain 1	Influences protein synthesis, growth, and apoptosis	Asian Descent	Meguro et al., 2010 [56]
*TBK1* ^1^	*rs12227270*	N/A	Essential role in regulation of inflammatory response	African Descent	Fingert et al., 2011 [64]
*TGFβR3*	rs1192415	Transforming growth factor (TGF)-β type III receptor	Binds TGF-β ligands	Asian Descent	Li et al., 2015 [65]
*TLR4*	rs2149356	Toll Like Receptor 4	Intracellular signaling of inflammatory pathways	Latin American	Navarro-Partida et al., 2016 [66]
*TLR4*	Asp299Gly	Toll Like Receptor 4	Intracellular signaling of inflammatory pathways	Latin American	Navarro-Partida et al., 2017 [67]
*TLR4*	Thr399Ile	Toll Like Receptor 4	Intracellular signaling of inflammatory pathways	Latin American	Navarro-Partida et al., 2017 [67]
*TMCO1*	rs4656461	Transmembrane And Coiled-Coil Domains 1	Regulates balance of calcium ions	European Descent	Burdon et al., 2011 [40]
*TMCO1*	rs7555523	Transmembrane And Coiled-Coil Domains 1	Regulates balance of calcium ions	European Descent	Bailey et al., 2016 [38]
*TMCO1*	rs28504591	Transmembrane And Coiled-Coil Domains 1	Regulates balance of calcium ions	African Descent	Bonnemaijer et al., 2018 [53]
*TMCO1*	rs7555523	Transmembrane And Coiled-Coil Domains 1	Regulates balance of calcium ions	Asian Descent	Hysi et al., 2014 [44]
*TMCO1*	rs7524755	Transmembrane And Coiled-Coil Domains 1	Regulates balance of calcium ions	Multi-Ethnic	Choquet et al., 2018 [45]
*TMTC2*	rs7961953	Transmembrane O-Mannosyltransferase Targeting Cadherins 2	Calcium homeostasis	Asian Descent	Nakano et al., 2009 [62]
*TMTC2*	rs324794	Transmembrane O-Mannosyltransferase Targeting Cadherins 2	Calcium homeostasis	Multi-Ethnic	Choquet et al., 2018 [45]
*TXNRD2*	rs35934224	Thioredoxin Reductase 2	Mitochondrial radical oxygen species scavenging	European Descent	Bailey et al., 2016 [38]
*TXNRD2*	rs16984299	Thioredoxin Reductase 2	Mitochondrial radical oxygen species scavenging	African Descent	Bonnemaijer et al., 2018 [53]
*ZP4*	rs693421	Zona Pellucida Glycoprotein 4	Component of the zona pellucida	Asian Descent	Nakano et al., 2009 [62]

^1^ Studies that specifically examined genetic associations with normal tension glaucoma.

**Table 2:** Genes associated with primary angle closure glaucoma. SNP: single nucleotide polymorphism.

Gene	SNP/Haplotype	Protein Product	Gene Function	Ethnicity	Citation
*ABCC5*	rs1401999	ATP binding cassette subfamily C member 5	Transport across plasma membrane	Asian Descent	Nongpiur et al., 2014 [142]
*ACVR1*	rs12997	Activin receptor type-1 protein	Bone morphogenic protein pathway signaling	Middle Eastern	Kondkar et al., 2020 [152]
*CALCRL*	AATACAGAT	Calcitonin Receptor Like Receptor	Transmembrane domain receptor activity	European Descent	Awadalla et al., 2012 [153]
*CHAT*	rs1258267	Choline O-Acetyltransferase	Production of acetylcholine in presynaptic terminals	Multi-Ethnic	Khor et al., 2016 [144]
*CHAT*	rs1258267	Choline O-Acetyltransferase	Production of acetylcholine in presynaptic terminals	Middle Eastern	Yousefian et al., 2020 [154]
*COL11A1*	rs3753841	Collagen Type XI Alpha 1 Chain	Produces component of type XI collagen	Asian Descent	Vithana et al., 2012 [145]
*COL11A1*	rs3753841	Collagen Type XI Alpha 1 Chain	Produces component of type XI collagen	European Descent	Vithana et al., 2012 [145]
*DPM2-FAM102A*	rs3739821	Dolichol phosphate-mannose (DPM) biosynthesis regulatory protein—Family with sequence similarity 102 member A	Regulates synthesis of DPM-role in estrogen activation	Multi-Ethnic	Khor et al., 2016 [144]
*eNOS*	Intron 4 VNTR repeat	Nitric oxide synthase 3	NO production	Middle Eastern	Ayub et al., 2010 [58]
*EPDR1*	rs3816415	Mammalian ependymin-related protein 1	Transmembrane protein for calcium-dependent cell adhesion	Middle Eastern	Yousefian et al., 2020 [154]
*EPDR1*	rs3816415	Mammalian ependymin-related protein 1	Transmembrane protein for calcium-dependent cell adhesion	Multi-Ethnic	Khor et al., 2016 [144]
*FERMT2*	rs7494379	Fermitin Family Member 2	Cell adhesion	Middle Eastern	Yousefian et al., 2020 [154]
*FERMT2*	rs7494379	Fermitin Family Member 2	Cell adhesion	Multi-Ethnic	Khor et al., 2016 [144]
*GLIS3*	rs736893	GLIS Family Zinc Finger 3	Transcription factor	Middle Eastern	Yousefian et al., 2020 [154]
*GLIS3*	rs736893	GLIS Family Zinc Finger 3	Transcription factor	Multi-Ethnic	Khor et al., 2016 [144]
*HGF*	rs5745718	hepatocyte growth factor	Regulates cell growth	Asian Descent	Awadalla et al., 2011 [141]
*HGF*	rs12536657	hepatocyte growth factor	Regulates cell growth	Asian Descent	Awadalla et al., 2011 [141]
*HGF*	rs12540393	hepatocyte growth factor	Regulates cell growth	Asian Descent	Awadalla et al., 2011 [141]
*HGF*	rs17427817	hepatocyte growth factor	Regulates cell growth	Asian Descent	Awadalla et al., 2011 [141]
*HSP70*	G+190C polymorphism	heat-shock protein 70	Protein folding processes	Middle Eastern	Ayub et al., 2010 [58]
*MMP9*	rs2250880	Matrix metalloproteinase-9	Regulates pathological remodeling processes	Asian Descent	Cong et al., 2009 [139]
*MMP9*	rs2664538	Matrix metalloproteinase-9	Regulates pathological remodeling processes	Asian Descent	Wang et al., 2006 [137]
*MMP9*	rs3818249	Matrix metalloproteinase-9	Regulates pathological remodeling processes	European Descent	Awadalla et al., 2011 [140]
*MMP9*	rs17576	Matrix metalloproteinase-9	Regulates pathological remodeling processes	European Descent	Awadalla et al., 2011 [140]
*MMP9*	rs17576	Matrix metalloproteinase-9	Regulates pathological remodeling processes	Middle Eastern Descent	Micheal et al., 2013 [155]
*PCMTD-ST18*	rs1015213	Protein-L-Isoaspartate (D-Aspartate) O-Methyltransferase Domain Containing 1-suppression of tumorigenicity 18	Protein repair/degradation – tumor suppressor	Asian Descent	Duvesh et al., 2013 [148]
*PLEKHA7*	rs11024102	Pleckstrin homology domain-containing family A member 7	Stabilizes E-cadherin junctions	Asian Descent	Vithana et al., 2012 [145]
*SMOC2*	rs13208776	SPARC-related modular calcium binding protein 2	Promotes matrix assembly	Middle Eastern	Al-Dabbagh et al., 2017 [161]
*SPATA13*	c.1432_1440del; p.478_480del	Spermatogenesis Associated Protein 13	Regulates cell adhesion and migration	European Descent	Waseem et al., 2020 [156]

**Table 3:** Genes associated with exfoliation (pseudoexfoliation) glaucoma. SNP: single nucleotide polymorphism.

Gene	SNP	Protein Product	Gene Function	Ethnicity	Citation
*AGPAT1*	rs3130283	1-acyl-sn-glycerol-3-phosphate acyltransferase alpha	Enzyme for lipid biosynthesis	Multi-Ethnic	Aung et al., 2017 [179]
*ATXN2*	rs7137828	Ataxin 2	Ataxin 2 production within cell cytoplasm	Asian Descent	Ma et al., 2019 [180]
*AVCR1*	rs12997	Activin A receptor, type I	Bone and muscle growth and development	Middle Eastern	Kondkar et al., 2020 [152]
*CACNA1A*	rs4926244	Calcium Voltage-Gated Channel Subunit Alpha1 A	Calcium ion transport	Asian Descent	Aung et al., 2015 [181]
*LOXL1*	rs1048661	Lysyl Oxidase Like 1	Connective tissue biogenesis	European Descent	Thorleifsson et al., 2007 [173]
*LOXL1*	rs3825942(G)	Lysyl Oxidase Like 1	Connective tissue biogenesis	European Descent	Thorleifsson et al., 2007 [173]
*LOXL1*	rs2165241	Lysyl Oxidase Like 1	Connective tissue biogenesis	European Descent	Thorleifsson et al., 2007 [173]
*LOXL1*	rs1048661	Lysyl Oxidase Like 1	Connective tissue biogenesis	African Descent	Williams et al., 2010 [171]
*LOXL1*	rs3825942(A)	Lysyl Oxidase Like 1	Connective tissue biogenesis	African Descent	Williams et al., 2010 [171]
*LOXL1*	rs3925942	Lysyl Oxidase Like 1	Connective tissue biogenesis	Asian Descent	Pandav et al., 2019 [182]
*LOXL1*	rs41435250	Lysyl Oxidase Like 1	Connective tissue biogenesis	Asian Descent	Ma et al., 2019 [180]
*LOXL1* ^1^	rs893818	Lysyl Oxidase Like 1	Connective tissue biogenesis	Asian Descent	Nakano et al., 2014 [183]
*LOXL1*	rs1048661(G)	Lysyl Oxidase Like 1	Connective tissue biogenesis	Middle Eastern	Abu-Amero et al., 2010 [184]
*LOXL1*	rs3825942(G)	Lysyl Oxidase Like 1	Connective tissue biogenesis	Middle Eastern	Abu-Amero et al., 2010 [184]
*LOXL1*	rs3825942(A)	Lysyl Oxidase Like 1	Connective tissue biogenesis	Middle Eastern	Asfuroglu et al., 2017 [185]
*LOXL1*	rs2165244	Lysyl Oxidase Like 1	Connective tissue biogenesis	Middle Eastern	Asfuroglu et al., 2017 [185]
*LOXL1*	rs41435250	Lysyl Oxidase Like 1	Connective tissue biogenesis	Latin American	Guadarrama-Valleji et al., 2013 [186]
*LOXL1*	rs1048661	Lysyl Oxidase Like 1	Connective tissue biogenesis	Latin American	Jaimes et al., 2012 [187]
*LOXL1*	rs216524	Lysyl Oxidase Like 1	Connective tissue biogenesis	Latin American	Takitani et al., 2018 [188]
*POMP*	rs7329408	Proteasome Maturation Protein	20S proteasome formation	Multi-Ethnic	Aung et al., 2017 [179]
*RBMS3*	rs12490863	RNA Binding Motif Single Stranded Interacting Protein 3	Tumor suppressor	Multi-Ethnic	Aung et al., 2017 [179]
*SEMA6A*	rs10072088	Semaphorin 6A	Actin cytoskeleton reorganization and central nervous system development	Multi-Ethnic	Aung et al., 2017 [179]
*TBC1D21* ^1^	rs16934339	TBC1 Domain Family Member 21	Mitochondrial structure	Asian Descent	Nakano et al., 2014 [183]
*TMEM136*	rs11827818	Transmembrane protein 136	Unknown	Multi-Ethnic	Aung et al., 2017 [179]

^1^ Nakano et al., 2014 identified 34 SNPs with varying association with *LOXL1*, *PML*, and *TBC1D21*. Only the most significant associations were reported in the table.

## Data Availability

No new data were created or analyzed in this study. Data sharing is not applicable to this article.

## Abbreviations

ABCC5	ATP binding cassette subfamily C member 5
ACD	anterior chamber depth
AD	African descent
ADAGES	African Descent and Glaucoma Evaluation Study
AUC	area under the curve
BMP	bone morphogenic protein
CCT	central corneal thickness
CI	confidence interval
CGSC	Chinese Glaucoma Study Consortium
DPM	dolichol phosphate mannose
ED	European descent
ECM	extracellular matrix
eNOS	endothelial nitric oxide synthase
GERA	Genetic Epidemiology Research in Adult Health and Aging
GLAUGEN	Glaucoma Genes and Environment
GST	glutathione S-transferase
GWAS	genome-wide association studies
HGF	hepatocyte growth factor
HSP70	heat-shock protein 70
HTG	high tension glaucoma
IOP	intraocular pressure
LAD	Latin American descent
LAZ	long anterior zonules
LOXL1	lysyl oxidase-like 1
MAF	minor allele frequency
ME	Middle Eastern
MFRP	membrane type frizzled related protein
NEIGHBOR	National Eye Institute Glaucoma Human Genetics Collaboration
NOS3	nitric oxide synthase gene
NTG	normal tension glaucoma
OR	odds ratio
PACG	primary angle closure glaucoma
PIC	plateau iris configuration
POAG	Primary open-angle glaucoma
PRS	polygenic risk scoring
RF	Risk factor
RNFL	retinal nerve fiber layer
SD	standard deviation
SNPs	single nucleotide polymorphisms
TGF-β	transforming growth factor beta
VCDR	vertical cup-disc ratio
WES	whole exome sequencing
XFG	exfoliation glaucoma
XFS	exfoliation (pseudoexfoliation) syndrome
